# Mechanism of chimera formation during the Multiple Displacement Amplification reaction

**DOI:** 10.1186/1472-6750-7-19

**Published:** 2007-04-12

**Authors:** Roger S Lasken, Timothy B Stockwell

**Affiliations:** 1 J. Craig Venter Institute, 9704 Medical Center Drive, Rockville, MD 20850, USA

## Abstract

**Background:**

Multiple Displacement Amplification (MDA) is a method used for amplifying limiting DNA sources. The high molecular weight amplified DNA is ideal for DNA library construction. While this has enabled genomic sequencing from one or a few cells of unculturable microorganisms, the process is complicated by the tendency of MDA to generate chimeric DNA rearrangements in the amplified DNA. Determining the source of the DNA rearrangements would be an important step towards reducing or eliminating them.

**Results:**

Here, we characterize the major types of chimeras formed by carrying out an MDA whole genome amplification from a single *E. coli *cell and sequencing by the 454 Life Sciences method. Analysis of 475 chimeras revealed the predominant reaction mechanisms that create the DNA rearrangements. The highly branched DNA synthesized in MDA can assume many alternative secondary structures. DNA strands extended on an initial template can be displaced becoming available to prime on a second template creating the chimeras. Evidence supports a model in which branch migration can displace 3'-ends freeing them to prime on the new templates. More than 85% of the resulting DNA rearrangements were inverted sequences with intervening deletions that the model predicts. Intramolecular rearrangements were favored, with displaced 3'-ends reannealing to single stranded 5'-strands contained within the same branched DNA molecule. In over 70% of the chimeric junctions, the 3' termini had initiated priming at complimentary sequences of 2–21 nucleotides (nts) in the new templates.

**Conclusion:**

Formation of chimeras is an important limitation to the MDA method, particularly for whole genome sequencing. Identification of the mechanism for chimera formation provides new insight into the MDA reaction and suggests methods to reduce chimeras. The 454 sequencing approach used here will provide a rapid method to assess the utility of reaction modifications.

## Background

Multiple displacement amplification (MDA) [1,2] is used to amplify plasmids [1], and BACs [3], and for whole genome amplification [2], for DNA from limiting samples [4], directly from small biological specimens [5], and from single bacterial cells for use in DNA sequencing [6]. MDA from single cells has enabled sequencing of novel microbial genomes, bypassing the need to develop culture methods [6-9]. The vast number of uncultured microbes in the environment are now amenable to sequencing using MDA from cells isolated by dilution or flow cytometry [6], micromanipulation methods [10,7,8] or microcolony technology [11].

One difficulty with MDA is its tendency to generate chimeric DNA rearrangements in the amplified DNA. For example, chimeras were found during sequencing in cloned libraries generated from MDA reactions [9]. The DNA rearrangements complicate genome assembly. While the correct sequence can be resolved by sequencing to a sufficient depth, it would be an important improvement to reduce chimeras, particularly considering the complexity of completing genomes of novel organisms. A high throughput method for sequencing organisms from environmental samples would be facilitated by elimination of the sequence rearrangements. Here, we have carried out an analysis of the chimeric sequences and the mechanism of their formation. The majority of chimeras were inverted sequences with an intervening deletion. The molecular mechanism that leads to the rearrangements was proven by sequencing 475 chimeric junctions generated by MDA.

## Results

**Figure 1:**
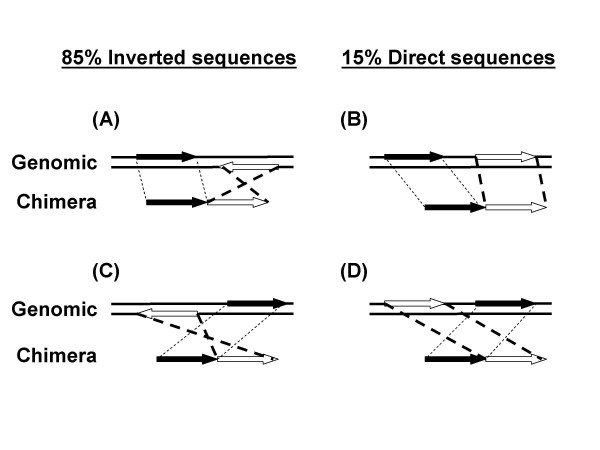
Types of chimeric rearrangements.

An MDA reaction from a single *E. coli *cell was analyzed by the 454 Life Sciences pyrosequencing method [12]. 495 chimeras were found in the 108,944 total uniquely mapped reads (10,878,753 total uniquely mapped bases) of *E. coli *K12 sequence. The chimeras were formed by the joining of two sequences. 475 chimeras could be unambiguously mapped to two genomic sequences (see Methods) and were included in the subsequent analysis of reaction mechanisms. In 406 chimeras (85%) a sequence inversion had taken place (Fig 1A and 1C; and Table 1). The second segment of the chimera was inverted from its original orientation in the genome. Only 69 (15%) of chimeras resulted from the joining of two segments in direct orientation (Fig 1B and 1D). The order of the two segments could also be reversed during the DNA rearrangement. That is, the first segment in the chimera (Fig 1, black arrows) could be joined to a segment that had been either downstream (Fig 1A and 1B, open arrows) or upstream (Fig 1C and 1D, open arrows) in the genomic sequence.

**Figure 2:**
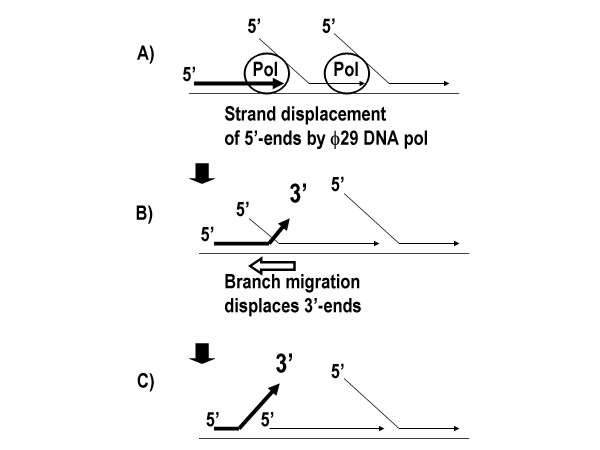
Mechanism of 5-end displacement by the DNA polymerase and 3'-end displacement by branch migration.

**Figure 3:**
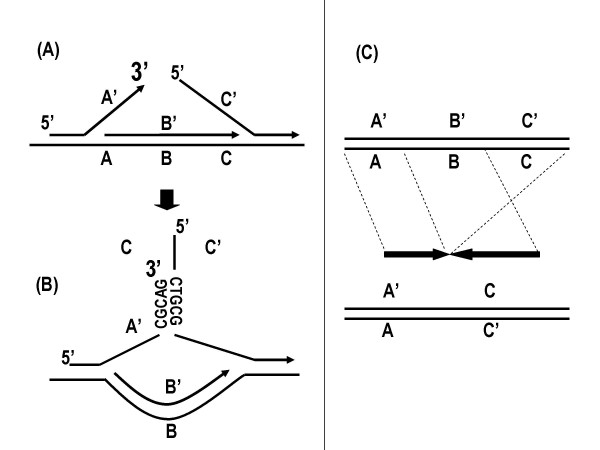
Mechanism of chimera formation with inverted sequences.

The rearrangements can be readily explained as occurring when displaced 3'-termini are freed to prime on nearby displaced 5'-strands. MDA occurs through a process where random hexamers prime multiple times on each template strand (Fig 2A). The reaction proceeds through a strand displacement mechanism with the phi29 DNA polymerase extending 3'-termini while concurrently displacing any downstream copies starting from their 5'-ends [1]. A branched DNA molecule results having numerous single stranded 5'-ends. Many of these are ultimately converted to double stranded DNA by the random hexamer primers. However, single stranded intermediate forms will be present and multiple alternative secondary structures are predicted to be stable. By a simple branch migration reaction, 3'-termini can be displaced (Fig 2B and 2C) and are available for mispriming events that would generate chimeras (Fig 3A and 3B). Branch migration is an energetically favorable mode of DNA strand exchange [13,14] with alternative forms predicted to occur in equilibrium.

**Figure 4:**
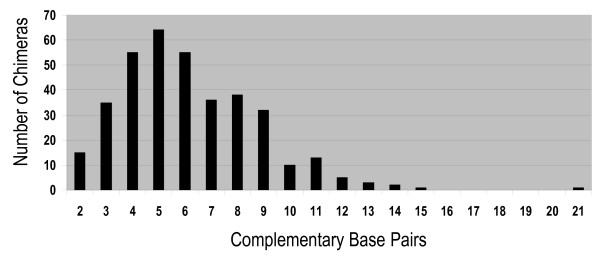
**Number of complimentary bases in the chimeric junction**. BLAST alignments of each of a chimera's segments to the *E. coli *genome revealed the junctions and the number of complimentary base pairs the 3'-end formed upon annealing to the new template (see Material and Methods).

The displaced 3'-terminus would be free to reanneal, preferentially at randomly occurring complementary segments on nearby 5'-strands (Fig 3B). The outcome will be the joining of two sequences in inverted orientation with an intervening deletion of the form A'C (Fig 3C). The finding that 85% of chimeras had this inverted form supports this as the likely mechanism. The chimeric junctions also reveal the site where the displaced 3'-end annealed to the second template and continued elongation. In the example (Fig 3B), from one of the sequenced chimeric junctions, priming was initiated on the new template where the sequence CGCAG-3' on the 3'-end had annealed to the sequence 5'-CTGCG-3' on the 5'-strand. In 76.8% of cases, the 3'-ends initiated priming at complimentary sequences of ≥ 2 bp on the new templates. These ranged up to 21 base pairs of complimentarity (Fig 4). The complimentarity occurred in 93.5% and 90.9% of cases for inverted and directly joined segments, respectively, when <10 kb apart, but only 34.8% and 27.6%, respectively, when >10 kb apart. Therefore, there was a significantly higher frequency of complimentary bases for the more proximal segments. 21 representative examples (from the inverted segments < 10 kb apart) are shown of the base pairs between the primer stand and new template at chimeric junctions (Table 2, bold nucleotides).

**Figure 5:**
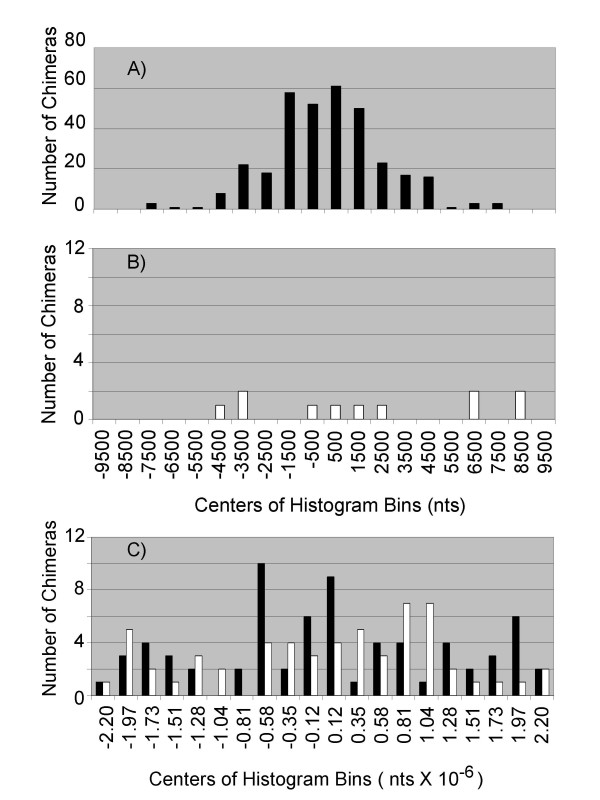
**Distances spanned by chimera junctions**. The size of the intervening genome segment spanned by each chimeric junction was sorted into one of twenty bins, and the resulting histograms plotted for A) inverted segments < 10 kb apart, B) Directly joined segments <10 kb apart, and C) inverted (closed bars) and directly joined (open bars) sequences for segments > 10 kb apart.

For inversions, the data is also consistent with a predominantly intramolecular mechanism in which a 3'-end relocates to a new template contained in the same branched DNA molecule (Fig 3A and 3B). The two segments joined tended to be less than 10 kb apart (Table 1) in the genome and would, therefore, frequently be contained in the same amplicon molecule. Furthermore, the distribution for inversions of segments <10 kb apart also supports an intramolecular mechanism where the limiting factor is the frequency of encounters between the 3'-end and new template. The number of inverted chimeras fell off with distance between the two segments (Fig 5A). If these segments were not contained in a single DNA molecule, no correlation to proximity in the genome would be predicted. However, the 3'-end could anneal to other molecules with no constraint on the map distance between the segments. Consistent with this, for segments >10 kb apart, the number of chimeras did not correlate to proximity (Fig 5C) for either inverted (closed bars) or direct (open bars) rearrangements. However, even when <10 kb apart, there was no apparent correlation to proximity for the few segments joined in direct orientation (Fig 5B). This is possibly due to a somewhat different mechanism involving hyperbranched DNA forms (see discussion).

**Figure 6:**
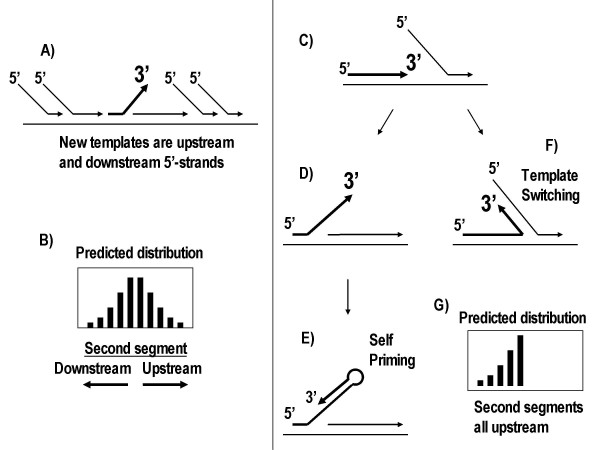
Alternate mechanisms of chimera formation.

Direct rearrangements result from the joining of two segments derived from the same genomic strand (Fig 1B and 1D). These were infrequent (69/475, Table 1) and had exactly the opposite pattern from inversions; less direct chimeras for segments <10 kb apart (Table 1, 11/69 = 16%), and more for >10 kb (Table 1, 58/69 = 84%). The model suggests, for the simplest branched amplicons (Fig 6A), that a displaced 3'-end has few opportunities to reanneal at a new location on the same template strand which would be mostly double stranded. This should disfavor intramolecular rearrangements. Annealing of the 3'-end could also occur on other DNA molecules which would present potential templates in both orientations. As predicted, these intermolecular events occurred at about the same rate for direct and inverted rearrangements (Table 1, 58 vs. 69 chimeras respectively) since the two genomic strands are equally represented among different DNA amplicon molecules. For these data, it can be calculated that inversions are favored over direct rearrangement in intramolecular reactions by about 26-fold ((58/69)(337/11) = 26).

The genomic order of the two segments was reversed (Fig 1C and 1D) in the chimera about half of the time for all chimeric forms (Fig 5A, B, C). Negative values indicate that the upstream segment in the chimera had begun as the downstream segment in the genomic sequence. As predicted from the model for inversions, the displaced 3'-end could anneal to new templates that would be available either upstream or downstream (Fig 6A). Also as predicted, for segments >10 kb apart there should be no preference (Fig 5C) for the order of segments since these are predominantly intermolecular mechanisms in which the 3'-end could anneal to any genomic location on the second molecule.

It was also possible to exclude two other potential mechanisms as playing a major role in chimera formation. It would be possible for the displaced 3'-end to form a hairpin structure and self-prime (Fig 6E). It would also be possible for the 3-end to begin priming on the same 5' strand that it had been displacing (Fig 6F). This kind of reaction, called template switching [14], is known to occur in some DNA replication reactions. While both of these mechanisms will result in inversions, the 3'-end can only extend on a new template that is upstream of it genomic sequence (Fig 6G – predicted histogram). Neither the hairpin nor the template switching mechanism can result in the 3'-end annealing to a new genomic location that is downstream. In contrast, the 3'-end is free to anneal to displaced 5'-strands that are either upstream or downstream (Fig 6A) with a predicted histogram (Fig 6B) that is born out by the experimental data (Fig 5A).

## Discussion

The mechanism of chimera formation by MDA was revealed by sequencing a whole genome amplification from an *E. coli *cell. 85% of the 475 chimeras evaluated were inverted sequences. MDA could produce these in a three step process; 1) initial extension of the random hexamer primers by strand displacement synthesis in which the phi29 DNA polymerase displaces 5'-ends, 2) displacement of extended 3'-ends by branch migration, and 3) mispriming of the 3'-ends on the nearby displaced 5'-strands. The first steps of random primer extension [1,2] and the displacement of 5'-ends [15,16] have been well established. The DNA polymerase could not displace the 3'-ends, however, these would be readily generated by a different mechanism involving branch migration. In MDA, multiple complimentary strands are concurrently synthesized from a template and these will compete for reannealing back to that template (Fig 2). Displacement of single stranded 3'-ends would be energetically favorable through a branch migration mechanism [13,14] with a resulting equilibrium between competing secondary structures. Displaced 3'-ends will be free to prime on new templates (Fig 3B) most frequently on nearby 5'-ends. We prove this model by showing that 85% of chimeras (Table 1) do indeed consist of an upstream sequence that has been extended on a second, nearby template of opposite polarity generating inversions (Fig 1). Moreover, the chimeric junctions show that in 76.8% of chimeras the upstream sequence had initiated priming on a short complimentary sequence in the new template (Fig 4, and Table 2). In the other 33.2% of chimeras priming did apparently occur with only 1 or no base pairs of complimentarity. This is not precluded since 3'-ends annealed transiently, even with some mismatches, would be rapidly stabilized as the polymerase extends them.

83% of inversions were formed by the joining of sequences that were less than 10 kb apart (Table 1, 337/406) in the genome consistent with an intramolecular process. This is reasonably consistent with the 12 kb average length of MDA products when denatured and resolved on an alkaline agarose denaturing gel [2]. These strands will be contained in larger branched and linear forms in the native state. Nevertheless, segments far greater than 10 kb apart would be less likely to occur in the same amplicon molecules.

All of the observations support intramolecular formation of inversions with rarer interaction between different amplicon molecules creating some direct and inverted rearrangements: 1) inversions are favored for segments that are <10 kb apart and more likely to be contained together in the same amplicon molecule, 2) all chimeric forms are infrequent for segments >10 kb apart (Table 1) since these would be intermolecular and diffusion limited, 3) inverted and direct rearrangements occur about equally for segments >10 kb apart because separate DNA molecules will contain potential single stranded templates of either strand equally, 4) within 10 kb, proximity of segments is favored for inversions (Fig 5A) agreeing with an entropic advantage that depends on containment of primer and template in one molecule, 5) direct rearrangements did not correlate to genomic proximity for segments <10 kb (Fig 5B) or >10 kb apart (Fig 5C, open bars) consistent with most being intermolecular processes, 6) simple branched amplicon molecules are predicted to contain displaced 3'-ends and an excess of displaced 5-strands of the polarity that would yield inversions (Fig 6A), and 7) direct joining for segments <10 kb apart (Fig 5B) is infrequent consistent with the lack of an obvious model for the reannealing of a 3'-end back to its own template at a new location. An intramolecular mechanism for direct rearrangements would be possible within a multiply branched DNA molecule, referred to as hyperbranched [16], where several rounds of replication had occurred. As newly synthesized strands serve, in turn, as templates for more synthesis and branching, displaced 5'-strands would be available in both polarities to serve as templates. However, this mechanism appears to have only generated a few chimeras. 11 of 475 chimeras (2.3%) were directly joined segments that were <10 kb apart (Table 1). This is more than predicted on a random basis ((2)(10 kb)/4.6 × 10^6 ^bp *E. coli *genome = 0.4%), but there was no apparent correlation to genomic proximity within the 10 kb set (Fig 5B). Perhaps highly branched molecules could more easily bring together the distal strands within single amplicon molecules.

About one chimeric junction was found per 22 kb of MDA generated DNA in the 454 sequences. A similar rate of chimera formation was found with the Sanger sequencing method using cloned libraries derived from MDA reactions [9]. 31.8% of clones (having an average of 3 kb inserts) were chimeric giving a frequency of about one rearrangement per 10 kb of MDA product. The authors hypothesized that single stranded DNA played some role in chimera formation and found that S1 nuclease treatment of MDA reactions, prior to use in cloning, dramatically reduced chimeras. Our work confirms the role of single stranded DNA as an intermediate in the pathway for chimera formation (Fig 2 and 3). It was also suggested that chimeras were somehow created by the library cloning process since they were not detected by PCR analysis in the original MDA reactions. Our data proves, in contrast, that the chimeras are created during the MDA reaction. Possibly the earlier work failed to detect chimeras in the MDA reaction because any particular junction sequence would be rare. S1 nuclease treatment of MDA amplicons should prove valuable for sequencing by the 454 method as well as from cloned DNA libraries as it should cleave the single stranded region that connects the two segments of the chimera (Fig 3B). This model also suggests why S1 nuclease does not eliminate all chimeras, leaving 6–8% of inserts still rearranged [9]. MDA generated chimeras that are eventually converted to the completely double stranded form would persist.

Over the past several years, MDA has enabled new experimental strategies in many research fields [4,6-8,17,18]. It has the potential to transform the field of metagenomics by allowing sequencing directly from cells isolated from the environment. Development of culture methods is no longer required in order to obtain sufficient DNA for sequencing. While the fidelity of the Phi29 DNA polymerase is very high [18,19], two notable alterations of the amplified DNA must be taken into consideration, amplification bias and chimeric rearrangements. While MDA is the least biased whole genome amplification method reported [2,5], there is some uneven representation over the genomic template. Bias was even greater when amplifying from single cells [6]. However, all genomic regions tested were represented, at least to some extent, and it was feasible to complete genomes simply by sequencing to a sufficient depth [9]. Chimeric sequences can also be resolved with sufficient sequencing depth, but add to the difficulty of assembling and closing genomes.

Understanding the mechanism of chimera formation should allow better optimization of MDA reaction conditions. It should be helpful to disfavor the annealing that occurs where displaced 3'-ends prime on new templates. The history of PCR development demonstrates many approaches for inhibiting nonspecific priming including lowering MgCl_2_, dNTP or DNA polymerase concentration, or increasing reaction temperature. Single strand DNA binding proteins recently introduced into MDA protocols [20] might also disfavor unwanted priming. Shorter MDA reaction times might also help depending on the dynamics of single stranded DNA accumulation and mispriming events. S1 treatment might also be more efficient at early MDA time points since chimeras are eventually converted to fully double-stranded DNA. Methods to reduce chimera formation are currently under investigation.

## Conclusion

The formation of chimeras is an important limitation to the MDA method. In the case of whole genome sequencing from single bacterial cells, it adds to the difficulty of the sequence assembly process. This is particularly important for novel organisms where the sequence had not been previously determined. Identification of the mechanism for chimera formation is a critical step in solving this obstacle and suggests many potential modifications to MDA that could reduce chimeras. The 454 sequencing approach used here will provide a rapid method to assess the utility of tested modifications.

## Methods

### Micromanipulation of a single *E. coli *cell

The system for micromanipulation has been described in detail elsewhere [10]. Briefly, an inverted microscope (Olympus IX70) with micromanipulation equipment (TransferMan NK2; CellTram Vario, Eppendorf) was used with sterilized glass capillaries (ID 10 μm) to isolate a single *E. coli *cell (strain K12, ATCC, catalogue number 19215) from a suspension of cells to 200 nl TE buffer.

### MDA whole genome amplification and 454 DNA sequencing

The single *E. coli *cell was placed on ice. 2.8 μl TE-buffer was added and MDA carried out in a 50 μl reaction volume using the REPLI-g kit (Qiagen) following the manufacture's recommended protocol. After incubation for 16 h at 30°C, the reaction was terminated at 65°C for 3 min. 3–5 μg of the MDA product was then used for 454-library construction and sequenced with the 454 Life Science GS 20 instrument according to the manufacturer's recommendations (454 Life Sciences, New Haven, CT).

### Informatics Analysis for chimera characterization

Reads were aligned to the *E. coli *K12 reference genome using GS 20 Mapping Software (454 Life Sciences, New Haven, CT). Reads categorized as PartiallyMapped were further analyzed by using NCBI BLAST Version 2.2.10 [21] to align each of the reads to the *E. coli *K12 reference sequence. Reads that had two segments of length >20 that mapped to noncontiguous portions of the reference genome were characterized as chimeric. Chimeric reads were further categorized based on the genome strand orientation of the pair of aligned read segments (they either map to the same genome strand or to opposite genome strands), the number of overlapping bases in the chimeric junction, and the size of the intervening genome segment spanned by the chimeric junction.

The chimera rate was calculated as the total of 495 identified chimeras divided by the number of base pairs sequenced. For 20 of these chimeras, the map location of one of the segments was ambiguous aligning to two or more different genomic regions. This reflects the repetitive character of the *E. coli *K12 genome sequence at small window sizes (20–50 nts) and the short 454 GS 20 read length of about 100 nts. Therefore, these 20 chimeras were omitted from the analysis of the mechanism of chimera formation which was carried out on the remaining 475 chimeras. The deleted segment refers to the nucleotides from the 3'-end of the first segment to the 5'-end of the second segment in the genomic sequence.

### Assignment of the chimeric junction and complimentary bases between the 3'-end and new template

The location of the chimeric junction was assigned from bases in common between BLAST alignments of each of the chimera's segments to the *E. coli *genome. Complimentary base pairs, where the displaced 3'-end annealed to the new template, were revealed by BLAST alignments where the first segment of the chimera had sequences in common with both genomic segments. In a few cases, BLAST had alternative possible junction assignments due to its use of sequence alignment scoring as opposed to thermodynamic favorability. However, analysis of sequence discrepancies suggests that BLAST was >95% accurate in assigning the correct nucleotide at which the 3'-end initiated priming on the new template.

## List of abbreviations

MDA: Multiple Displacement Amplification

## Authors' contributions

RL provided the MDA whole genome amplification from a single *E. coli *cell, obtained 454 sequencing data, proposed the reaction mechanisms for chimera formation and drafted the manuscript. TS determined the sequences of the chimeric junctions, analyzed them for strand polarity and distance between joined segments, and noted the complimentary bases shared between the two segments. All authors read and approved the final manuscript

## Figures and Tables

**Table 1:** Types of chimeric DNA rearrangements

	Distance between joined segments^1^	Number of chimeras
Inverted Sequences	< 10 kb	337
	> 10 kb	69
**Total**		**406**
Direct Orientation	< 10 kb	11
	> 10 kb	58
**Total**		**69**
**All forms**		**475**

^1^The distance (nucleotides) in the original genomic sequence, between the 3' end of the first segment and 5' end of the second segment to be joined in the chimera.

**Table 2:** Representative examples of complimentary bases between the displaced 3'-end (upper sequence) and the new template (lower sequence).

ReadID	
075540_3218_3423	5'-CCGAAAATTACTCT**ACC**-3'
	3'-**TGG**CTGTTACTGAGCGCTTAGGTCGT-5'
116645_2999_1485	5'-ATAAATTAAATGA**ATG**-3'
	3'**TAC**TCGAAAGGAGTGCTGACCCAGCCCG-5'
011550_4060_0841	5'-ACCACAACAGAACCTT**CTTT**-3'
	3'**GAAA**CTTTTACTGTTGTAGTAAGTG-5'
045228_3788_1694	5'-CCTCGTCGG-CGGTTTT**CATC**-3'
	3'-**GTAG**AC-CTTACACGTTTTACCG-5'
045274_2234_1769	5'-TACGGTTGTATGC**TGCGC**-3'
	3'-**ACGCG**AACAGATATCCACCTCAAATGC-5'
127787_3000_2253	5'-CCAGCAGGGATTGCGTCA**TCGC**-3'
	3'-**AGCG**TATTGGGCATGGTTTCAGT-5'
106844_3069_3603	5'-ATGTAGCCAGCAA**CATTAT**-3'
	3'-**GTAATA**TTCGTTCTCGGTATGGGTTCTC-5'
002131_3149_2822	5'-GGCGACTATGCACTA**GGGAA**-3'
	3'-**CCCTT**ACGGACACGTTTAATCAAGAC-5'
175043_3546_0829	5'-ATCTGATAGTCCAGA**AAACC**-3'
	3'-**TTTGG**TCGTAGTTGTTTCTCTAACCA-5'
114939_3307_2524	5'-CTTCGACGATACCCATG**CCGGA**-3'
	3'-**GGCCT**TCTTCACGCATTTTGCTTGA-5
148627_3831_3379	5'-CCCATTGCCGTCGTACTTTTGA**TTGTT**-3'
	3'-**AACAA**TGAAGCCCGTTTCC-5'
004326_2840_3105	5'-CGCTGCGTGATTCCCA**CGCCGG**-3'
	3'-**GCGGCC**ATGCCTTTGG-CTTAACAG-5'
096255_3814_0862	5'-CCAGTGAATTTCACTTCG**CCAACG**-3'
	3'-**GGTTGC**CCGACCGGGTGTTCAAC-5'
034769_3258_1412	5'-TGTACTAAAAGGGTAGTC**AGAAAAA**-3'
	3'-**TCTTTTT**GGTCCAGAGCTAAAAT-5'
151610_2448_0137	5'-GAGGCAACATTTGATC**GTCAGT**G-3'
	3'-**CAGTCA**TACTTTTCAGGCACCGTCG-5'
066589_3139_3159	5'-CGCCAGGAAACATTGCA**CACC**A**CGC**-3'
	3'-**GTGG**G**GCG**CTAGCGCTCCGTTTGG-5'
173672_2559_2768	5'-CATTCCCGGAATTAC**ATATCTTT**-3'
	3'-**TATAGAAA**AAGTAATCCGTCACCGGA-5'
116413_2206_3333	5'-GCATATCTCCATCCT**GAGT**G**ACGC**-3'
	3'-**CTCA**T**TGCG**AAAACCAACCCGCTCTT-5'
117929_3656_1113	5'-TTTGAAATATCCACTATTAAGCTA**GTGTTTAACG**-3'
	3'-**CACAAATTGC**GTCGGAA-5'
058603_2536_1539	5'-CCGCATGCATGACC**GTTTTTGTGAA**-3'
	3'-**CAAAAACACTT**AGAGTGGTTAGGGTTT-5'
127287_2649_1441	5'-GCGCACGTAGTTC**GCAGGACGC**-**GGGT**-3'
	3'-**CGTCCTGCG**G**CCCA**AGACGACTAGCTAT-5'

All examples are from the set of inverted chimeras for segments <10 kb apart. Base pairs between the 3'-end and the new template are shown in bold. (see Methods for Assignment of Chimeric Junctions).
